# Supplementary material for: Are carbon emissions trading and green financial instruments synergistic? -Comprehensive quantitative research based on content analysis
Source: PLoS One. 2024 Mar 7;19(3):e0298601. doi: 10.1371/journal.pone.0298601 (PMC10919601; doi:10.1371/journal.pone.0298601)
Supplement: S1 Data — (ZIP) [file pone.0298601.s001.zip › Data/地级市Z_F工作报告.zip!/╒π╜¡/║╝╓▌╩╨/└┤╫╘╣1⁄2╓┌║┼í╛┬φ┐╦╩2╛▌═°í┐_2018.docx]

各位代表：

现在，我代表市人民政府向大会报告工作，请予审议，并请市政协委员和其他列席人员提出意见。

一、2017年工作回顾

2017年是新一届市政府开局之年,也是实施市“十三五”规划重要一年。市政府认真学习贯彻习近平新时代中国特色社会主义思想，在省委省政府和市委的正确领导下，紧紧依靠全市人民，按照“秉持浙江精神，干在实处、走在前列、勇立潮头”的要求，统筹谋划抓开局，求真务实抓落实，较好完成了市十三届人大一次会议确定的年度目标任务，全市经济社会保持了健康发展的好势头。我们朝着新目标，迈出新步伐，取得了新业绩。

——经济质效持续提升。全市生产总值达到12556亿元，增长8.0%。财政总收入2921.3亿元，增长14.2%；其中，一般公共预算收入1567.42亿元，增长17.4%。社会消费品零售总额5717亿元，增长10.5%。全社会固定资产投资完成5857亿元，增长1.4%，其中民间投资3301亿元，增长10.4%。货物进出口总额5085亿元，增长13.3%；其中，进口1629亿元，增长38.8%；出口3456亿元，增长4.3%；跨境电商出口70.22亿美元，增长15.9%。全市三次产业结构调整为2.5：34.9：62.6；信息经济增加值3216亿元，增长21.8%,对经济增长贡献率超50%。规模以上工业企业和服务业企业利润分别增长7.7%和26.3%，经济运行质量进一步提高。

——重点改革进展明显。全市“最多跑一次”实现事项9593项，其中市本级771项；公布凭居民身份证即可办理的事项296项；不动产登记实现全流程60分钟领证，商事登记新设企业85%可按“一件事”标准网上办理，投资项目审批周期再提速。整合设立市数据资源管理、投资促进、外事侨务、行政复议等机构；萧山余杭富阳与杭州主城区社保一体化正式运行，临安撤市设区。

——创新动力有效增强。规上工业科技活动经费支出总额297亿元，增长24.3%；全社会研发经费支出与地区生产总值之比预计提高到3.2%。新增发明专利授权量9872件，增长14.2%。新入选“国千”人才16名、“万人计划”人才18名、“省千”人才51名。引进外籍人才6150名、海归人才4068名。接收应届高校毕业生7.93万名；其中，研究生以上学历1万名，增长20.9%。新增国家重点扶持高新技术企业589家、境内外上市企业28家、新三板挂牌企业34家。新设各类市场主体20.02万户，市场主体总量突破百万户。

——城市建设成效显著。交通基础建设快速推进，地铁2号线钱江路站-良渚站、4号线近江站-浦沿站建成运营，新增运营里程35.8公里。城市快速路开工64.8公里、续建35.1公里。城中村改造规模大、进度快、质量好， 69个村完成整村征迁；小城镇环境综合整治完成52个乡镇。

——环境质量持续改善。全市9个县控以上劣Ⅴ类水断面、1256个劣Ⅴ类水体如期完成“剿劣”任务；市控以上断面达到或优于Ⅲ类水标准的比例上升3.8个百分点，提高到88.5%；杭州与周边城市交接断面水质获省级考核优秀。空气优良天数同比增加11天；PM2.5平均浓度为每立方米44.6微克，下降8.6%。成功创建国家生态园林城市,获得美丽山水城市称号。

——民生事业不断发展。城镇居民、农村居民人均可支配收入分别增长7.8%和8.9%。新增城镇就业29.71万人，失业人员再就业11.04万人，城镇登记失业率1.7％。开工安置房项目51个，建筑面积693万平方米；竣工50个，面积582万平方米。完成城镇危旧房治理改造18.1万平方米、农村困难家庭危房改造1796户，地质灾害隐患点消除397个。获得全国文明城市“三连冠”，蝉联中国最具幸福感城市称号。

过去一年，我们全面贯彻新发展理念，针对新情况新问题，抓重点、攻难点、解热点，着力打好环境整治、交通建设、平安创建等“六场硬仗”，主要做了以下工作：

（一）抓改革、增活力，优化发展环境。我们突出群众满意导向，优化办事流程，全面推进“最多跑一次”改革。商事登记改革率先在全国推出“1+N”+X多证合一、证照联办和“商事登记一网通”措施；全面启动投资项目在线审批监管平台2.0版，并在模拟审批、施工图联审、涉审中介治理等关键环节取得突破；公民个人事项“简化办、网上办、就近办”扎实推进，全面实施便民服务事项办理双休日开放。坚持“无条件归集、有条件使用”的原则，累计归集59个部门289.13亿条政务数据，打破信息孤岛取得实质性进展，市统一政务咨询投诉举报平台整合工作有力推进。积极支持桐庐县开展“最多跑一次”改革与法律法规衔接省级试点。贯彻“房子是用来住的、不是用来炒的”定位，优化综合调控措施，积极探索建立租购并举的住房体系，出台主城区商品住宅出让用地配建公租房不低于总建筑面积10%等政策，首次实施了人才租赁房用地公开出让，成为全国首批住房租赁试点城市，房地产市场平稳有序发展。深入推进供给侧结构性改革，淘汰落后和过剩产能170家（项），整治提升“低小散”企业3753家，处置“僵尸企业”65家；出台减负降本“28条”等政策，为企业减负594亿元；消化批而未供土地3.4万亩、利用供而未用土地5.1万亩、推进低效用地再开发3.5万亩。深化农村改革，推进农村土地承包权确权登记，深化村级集体经济股份制和农村“三位一体”改革。综合行政执法改革实现了区县（市）全覆盖。交通管理体制改革基本完成。深化户籍制度改革，全面实施新型居住证制度，制定出台居住证积分管理办法。加强信用杭州建设，成为全国首批社会信用体系建设示范城市。支持设立了全国首家互联网法院，对互联网经济发展和消费者维权加强司法保障。

（二）强创新、添后劲，转换发展动能。我们深入贯彻实施创新驱动发展战略，城西科创大走廊重要基础设施建设全面推进，之江实验室落地，阿里达摩院成立，创新要素加快集聚；钱塘江金融港湾实施意见出台，重点项目建设实质性推进；城东智造大走廊发展规划完成编制，进入启动建设阶段。杭州高新区在147个国家级高新区综合排名中跃居第三。国家级杭州临空经济示范区获批设立。杭州经济开发区、万向集团、浙江大学成为第二批国家双创示范基地，白马湖生态创意城入选首批国家级文化产业示范园区，玉皇山南基金小镇、梦想小镇被命名为首批省级特色小镇。新增国家级、省级众创空间20家和57家；新增国家级、省级孵化器2家和11家，新认定省级企业研究院76家。深入实施科技企业“双倍增”行动计划，新增省科技型中小企业1688家、市重点培育科技型初创企业506家。组织实施“凤凰行动”计划，大力推进企业上市和并购重组，境内上市企业数居全国大中城市第四。加强人才工作，出台实施新引进高学历毕业生生活补贴发放、人才国际化等新政，打响海外引才引项目“一会一赛”品牌，人才流入率和海归人才净流入率居全国城市首位，入选“外籍人才眼中最具吸引力的中国城市”前三位。全国首个国际人才创业创新园挂牌，杭州国家自主创新示范区外国人签证、停居留等七项出入境便利政策获公安部批准落地。深入实施“一号工程”，电子商务、信息软件、大数据云计算等优势产业继续保持高速增长，装备制造业、高新技术产业、战略性新兴产业增长快于传统工业，人工智能、虚拟现实等未来产业蓄势初发。制定出台改造提升传统制造业实施方案，开展萧山化纤、富阳造纸两个省级分行业改造提升试点。推进“两化”深度融合，持续实施智能制造等专项行动，新增“企业上云”4.15万家，完成“机器换人”重点项目574个、“工厂物联网”项目123个。抓好招商引资工作，全年利用外资66.1亿美元，招引内资1562.8亿元，浙商回归到位资金761.8亿元。加快外贸动能转换，服务贸易创新发展。加快发展电子商务，着力扩大消费。推动会议展览业加快发展，承办了金砖五国配套会议、中国—中东欧国家文化合作部长论坛等高端国际会议，“一带一路”地方合作委员会秘书处、世界旅游联盟总部、国际标准化组织会议基地、中国国际茶叶博览会等国际组织和重要会议落户杭州。推进旅游国际化、全域化，旅游总收入突破3000亿元、增长18.3%，入境游客突破400万人次、增长10.7%，获得中国旅游休闲示范城市、世界旅游组织“全球15个旅游最佳实践样本城市”称号。农业“两区”加快建设，新建“菜篮子”基地5607亩，农林牧渔业增加值增长2.0%，现代农业稳步发展。

（三）重谋划、促建设，提升基础设施水平。我们针对交通基础设施等方面存在的短板，深入谋划和加快建设城市轨道交通、快速路、高速公路、高铁、机场等现代化交通基础设施。制定实施《杭州市城市轨道交通建设五年攻坚行动计划》，推进市轨道交通办公室实体化运作，完善地铁筹资机制，地铁二期加快建设，地铁三期各项前期进展顺利。制定出台《杭州市“迎亚运保畅通”城市快速路网建设四年攻坚行动计划》，紫金港立交等工程建成通车，钱塘江博奥隧道开工建设，望江路隧道、文一路地下通道等项目稳步推进。《杭州铁路枢纽规划(2016-2030年)》获批，铁路杭州西站完成站点选址及周边规划研究，铁路杭州南站建成，杭黄铁路杭州段主体工程全线贯通。萧山机场新开辟国际航线15条，旅客吞吐量增长12.6%。杭州绕城高速公路西复线、临金高速公路国高网杭州段完成年度建设任务，千黄高速公路淳安段开工建设，京杭运河三级航道整治工程杭州段加快推进。“城市数据大脑”试点进展顺利，主城区公交分担率继续提高，新建停车泊位5.69万个，新增（扩容）公共自行车服务点123个，市区交通拥堵持续缓解。污水管网新增393.86公里，雨水管网新建145.7公里、提标改造32.86公里，七格污水处理厂四期、临平净水厂等项目加快推进。供水管网新建173.95公里、改造111.13公里。千岛湖配供水工程建设加快推进。深入推进“五废共治”，九峰环境能源项目、萧山临浦和钱江餐厨废弃物资源化利用项目建成试运行。制定亚运会场馆建设计划，第一批33个场馆设施及亚运村项目建设全面启动。竣工地下空间 701.8万平方米，全面启动艮山路、德胜路、沿江大道、大江东等综合管廊项目建设。成为首批国家装配式建筑示范城市。

（四）攻难点、优环境，改善城乡面貌。我们坚持块抓条保、上下联动，全面推进城中村改造、小城镇环境综合整治，深入推进“三改一拆”。完成“三改”3077.1万平方米，拆除违法建筑2516.9万平方米，拆后利用率79.3%。坚决打好劣Ⅴ类水剿灭战，深入推进工业污染、农业面源污染等专项治理，完成截污纳管项目356个、雨污分流项目349个，河道清淤1254万方、综合整治281.9公里。钱塘江杭州段全面禁止采砂。持续开展大气治理，启动全市域“清洁排放区”建设，组织实施减排项目124个，全面完成热电锅炉、工业锅炉清洁化改造；淘汰老旧汽车23119辆，新增清洁能源和新能源公交车879辆；削减挥发性有机物排放量9974吨。深化垃圾分类、清洁直运、末端处置工作，制定实施源头分类减量、处置能力建设两个三年行动计划。市区全年新增绿地487万平方米。推进新一轮区县（市）协作、联乡结村工程，实施协作项目84个，投入4亿元。成为首批国家历史建筑保护利用试点城市。继续实施古村落保护工程，启动第二批“杭派民居”示范村创建工作，创建美丽乡村精品示范线12条。加强农房建设管理，规范农村宅基地管理。农村土地综合整治试点为乡村振兴找到一条好的路子。

（五）保民生、提品质，加强公共服务。我们坚持以人民为中心的发展思想，着力抓好民生保障和社会治理工作。年度10件民生实事项目全部完成。全市基本养老、医疗保险的参保率分别达95%、98%以上。坚持创业带就业，出台实施新一轮大学生创业三年行动计划，加强高校毕业生、就业困难人员、农村转移劳动力等重点人群就业服务，现代学徒制国家级改革试点扎实推进。向全市低保家庭、残疾人基本保障对象、低保边缘家庭18.7万人发放救助金7.77亿元。推出配租房源8514套，保障住房困难家庭1.02万户。深入实施新名校集团化战略，推进市域优质教育资源共建共享；中小学（幼儿园）竣工66所、开工建设133所，3个区县新创建成为省基本实现教育现代化县（市、区）。杭师大入选省第二批重点建设高校，浙大城市学院怀卡托大学联合学院成立并招生，浙江西湖高等研究院招生。文化建设和文艺创作蓬勃发展，1部作品和 14部作品分别入选全国、全省“五个一工程”，舞剧《遇见大运河》海内外巡演获得好评，大型水上情景表演音乐会《最忆是杭州》成功复演；新建农村文化礼堂150家、社区文化家园50家。圆满完成第十三届全国学生运动会服务保障任务。成功举办市第十九届运动会。良渚古城遗址申遗步伐加快。城市档案中心等公共设施建设项目稳步推进。“双下沉两提升”、“三医”联动、“分级诊疗”等综合医改深入推进，医养护一体化家庭医生签约人数达283.9万，智慧医疗应用在省市县三级公立医院及社区卫生服务机构实现全覆盖，有效防范了登革热疫情的蔓延。完成全国和全省养老服务综合改革试点任务，社区养老、医养结合等取得明显成效。强化安全生产责任体系建设，各类生产安全事故数、死亡人数、受伤人数分别下降17.8%、19.4%、15.7%。持续打击“盗抢骗”“黄赌毒邪”和环境、食品药品等领域违法犯罪行为。积极防范化解企业“两链”风险等金融风险。既有住宅加装电梯取得实质性突破。持续推进城乡社区治理和服务创新，获得“法治政府建设典范城市”称号。较好完成对口支援和东西部扶贫协作、山海协作等任务。国防动员和后备力量建设、双拥优抚、外事、港澳台侨事务、民族宗教、地方志、气象、残疾人、慈善、红十字、老龄、妇女儿童、关心下一代等工作，都取得新的进展。

过去一年，市政府高度重视政治建设，坚持正确政治方向，牢牢把握政治原则，牢固树立“四个意识”，严格履行主体责任，切实落实全面从严治党要求，不断强化自身建设。严格依法行政、努力高效施政，推进政府法律顾问全覆盖，提请市人大常委会审议地方性法规6件，制定和修改政府规章6件，办理人大建议和政协提案1083件，回复市人大常委会审议意见书15件。加强财政资金使用管理，强化审计监督，加强廉政建设，进一步营造风清气正的政务环境。

各位代表，我们所取得的成绩来之不易，最根本的是靠以习近平同志为核心的党中央的坚强领导！同时，这是省委省政府和市委正确领导的结果，是市人大、市政协监督支持的结果，是全市广大干部群众共同奋斗的结果！在此，我代表市人民政府，向全市人民和外来建设者，向市人大代表和政协委员，向各民主党派、工商联、无党派人士、各人民团体和社会各界人士，向中央驻杭单位和省级各部门，向驻杭解放军和武警部队官兵，向关心支持杭州发展的港澳台同胞、海外华人华侨和国际友人，表示衷心的感谢、致以崇高的敬意！

我们也清醒看到杭州发展中存在的不平衡、不充分问题，主要有：科技创新和人才支撑力还不够强，传统产业转型升级有待加快；交通、环保等基础设施短板需要加快补上；城市国际化步伐尚需加快，城乡发展差距有待进一步缩小；公共服务供给有待加强，城市安全运行还存在压力，社会治理现代化水平还需提高。政府工作也存在不足，一些政府机关工作人员知识不足、能力不足问题客观存在，一些领域腐败问题仍有发生。对此，我们将直面问题，敢于担当，尽心竭力加以解决。

二、当前形势和2018年重点工作

党的十九大以习近平新时代中国特色社会主义思想为指导，明确了新时代中国特色社会主义发展的战略安排和总体部署，开启了决胜全面建成小康社会、建设社会主义现代化国家的新征程。省委十四届二次全会作出了《关于高举习近平新时代中国特色社会主义思想伟大旗帜奋力推进“两个高水平”建设的决定》，对我省我市发展提出了新的更高要求。市委十二届三次全会明确了新时代杭州“三步走”奋斗目标：到2020年，高质量完成“十三五”规划任务，高水平全面建成小康社会，成为具有较高全球知名度的国际城市；到2035年，社会主义现代化目标基本实现，成为特色彰显、具有较大影响力的世界名城；到本世纪中叶，社会主义现代化建设走在全国前列，成为具有全球重大影响力的独特韵味别样精彩世界名城。

我们要准确把握杭州今后工作的总体要求：高举习近平新时代中国特色社会主义思想伟大旗帜，深入学习贯彻党的十九大和中央经济工作会议精神，认真贯彻落实省党代会、省委十四届二次全会和市党代会、市委十二届三次全会部署，坚持稳中求进工作总基调，坚持新发展理念，坚持高质量发展根本要求，聚焦社会主要矛盾抓重点、补短板、强弱项，坚定不移推进城市国际化、坚定不移推进拥江发展、坚定不移推进创新驱动发展、坚定不移推进城市治理体系和治理能力现代化、坚定不移全面深化改革，以一流状态建设一流城市，不断厚植创新活力之城、历史文化名城、生态文明之都、东方品质之城特色优势，继续朝着建设独特韵味别样精彩世界名城目标迈进，在全省“两个高水平”建设中更好发挥龙头领跑示范带动作用。

面对新目标、新要求，我们要围绕满足人民对美好生活的需要，坚持高质量发展根本要求，自觉遵循自然规律、经济规律、社会规律、城市发展规律，正确处理重点与一般、发展与民生、近期与长远的关系，以筹备召开2022年亚运会为重要节点目标，打好“最多跑一次”改革、环境整治提升、重大基础设施项目建设、重大产业项目推进、民生实事推进、防范化解风险等新“六场硬仗”，保持定力、积极有为，脚踏实地、久久为功，推动经济社会发展和各项工作不断迈上新台阶。发展要重创新，进一步提升经济质量。从打造新经济重要策源地、引领全国的创新型城市、长三角创新发展新引擎的高度，以供给侧结构性改革为主线，强化新动能培育和传统动能修复，高水平打造以数字经济为核心、新经济为引领、传统优势产业为支撑的现代化经济体系。把高质量要求贯穿于各方面工作的理念、思路、政策和举措中，努力形成推动高质量发展的制度体系，加快质量变革、效率变革、动力变革，勇当全国全省高质量发展的排头兵。改革要重突破，进一步激发市场活力。从治理体系和治理能力现代化的高度，强化法治建设，坚持问题导向、先行先试、制度创新，以深化“最多跑一次”改革为突破口，努力在政府自身改革、经济体制改革、城市建设管理体制改革等方面取得更大的进取突破，使市场在资源配置中起决定性作用，更好发挥政府作用，持续激发市场活力和社会创造力。建设要重推进，进一步增强城市能级。从加快建设现代化城市的高度，以筹备亚运会为契机，全面提升交通等基础设施水平，积极推进“拥江发展”，加快城市国际化和城乡一体化，主动参与全省大湾区大花园大通道大都市区建设，深度对接“一带一路”、长江经济带、长三角城市群建设，增强城市辐射带动能力，推动形成全面开放新格局。民生要重品质，进一步增进群众福祉。从践行以人民为中心发展思想的高度，聚焦重点持续发力，坚决打好防范化解重大风险、精准帮扶、污染防治的攻坚战；聚焦城乡融合、乡村振兴、区域协调，加快农业农村现代化；聚焦事关群众切身利益的“关键小事”，尽力而为、量力而行，精准施策、靶向操作，守住底线、筑牢底部，加强民生保障和公共服务供给，使群众获得感、幸福感、安全感更加充实、更有保障、更可持续。

2018年是贯彻党的十九大精神开局之年，是实施“十三五”规划、决胜高水平全面建成小康社会的关键一年。全市发展主要预期目标是：地区生产总值增长7.5%左右，一般公共预算收入增长7.5%，研发经费支出与地区生产总值之比达3.3%左右，城乡居民人均收入与经济增长基本同步，城镇新增就业人数和节能减排指标完成上级下达任务。

重点做好六个方面工作：

（一）聚焦“最多跑一次”改革突破口，着力提高改革开放水平。今年是改革开放40周年，我们要以“最多跑一次”改革为牵引，进一步撬动重点领域关键环节改革向纵深推进，构建对外开放新格局，持续增强发展活力和动力。

推动“最多跑一次”改革持续突破。以打造“移动办事之城”为目标，优化公共数据大平台建设，深入推进系统互通、数据共享，全面推行政务服务事项网上办。整合优化业务办理流程，实现“一窗受理、集成服务”，力争“最多跑一次”改革事项全覆盖。实施“证照分离”改革，优化商事登记“一网通”平台，实现多证合一与证照联办事项全覆盖。全面实施并不断完善市场准入负面清单制度。继续优化投资项目审批流程，推进企业投资项目承诺制改革和“网上中介超市”建设，一般企业投资项目开工前审批“最多100天”。全面推行不动产登记“一个系统、一份材料、一个流程”办理，扩大“60分钟办结”受益面。推广移动办事终端，推动群众事项办理向乡镇（街道）延伸，努力实现个人办事“移动可办”“就近能办”。在政务服务“一次办结”的同时，完善咨询投诉“一号响应”，探索市场监管、环境保护、安全生产、文化等领域执法监管“一次到位”。

深化要素配置市场化改革。各行业各领域要全面贯彻节约集约用地的方针，立足于盘活存量土地，提高土地利用效率，实现土地利用方式根本性转变。深入推进土地整理、水田垦造，加强耕地保护，提高土地质量。着力盘活利用批而未供土地、开发建设供而未用土地，加大力度推进低效用地再开发。推进“标准地”改革，对工业、商业和旅游用地，探索实行带能耗、环境、建设、“亩产”等标准出让。完善土地出让办法，优化开发建设条件约束。深化实施“凤凰行动”计划，大力支持企业上市、并购重组，新增上市企业15家以上，培育一批在全国有重大影响力的龙头企业。强化政府引导，更好发挥各类产业基金的引导和放大作用，推动金融与科技、产业、经济良性互动。以打造国际金融科技中心为目标，支持新金融规范发展，加快钱塘江金融港湾建设。

激发各类市场主体活力。深化国资国企改革，稳妥推进混合所有制改革，组建国有资本运营公司，做强做优做大国有资本。推进国资重组和布局优化，推动国有资本参与战略投资，助推全市重点产业发展、重大科技研发、重大基础设施建设。优化信用环境、法治环境、政务环境、营商环境，支持民营企业创新发展，有效激发民企活力。构建“亲”“清”新型政商关系，更好地营造企业家健康成长的环境，大力弘扬杭商精神，充分发挥企业家作用。

提升对外开放和城市国际化水平。对接“一带一路”建设，深化跨境电商综试区建设，加快推进全球电子商务平台（eWTP）杭州实验区建设，努力建设“数字丝绸之路”重要战略枢纽城市。大力引进外资，鼓励企业“走出去”，培育外贸新业态，加快服务贸易创新发展。对接中国国际进口博览会，扩大先进设备和优质产品进口。提升杭州与港澳台合作水平。深化西博会和休博会转型升级，提升文博会、茶博会、电博会、动漫节、云栖大会、国际人才大会等重大会展影响力，积极引进高端国际会议展览和国际组织，打响国际会展之都品牌。深入推进旅游国际化、旅游全域化，做好世界旅游产业博览会、2018中美旅游高层对话等重大活动的服务保障工作。深化文化对外交流，推进东方文化国际交流重要城市建设。

（二）聚焦创新驱动和结构优化，着力推动经济高质量发展。以建设现代化经济体系为目标，以建设国家自主创新示范区和创建“中国制造2025”国家级示范区为载体，坚持质量第一、效益优先的导向要求，深化供给侧结构性改革，突出新经济引领，强化新动能培育和传统动能修复，加快推动实体经济、科技创新、现代金融、人力资源协同发展。

科技创新是杭州发展的显著特征和第一动力。要紧紧抓住科技创新“牛鼻子”，坚持“企业主体、人才引领、平台保障”，推动科技与产业紧密结合，加快创新驱动发展。充分发挥企业主体作用。深入实施创新型企业、高新技术企业和科技型中小企业培育工程，支持企业加大研发投入，深化产学研合作，积极创建国家和省级制造业创新中心，新建市级以上制造业创新中心3家以上，新认定市级以上企业技术中心30家以上。实施“专精特新”“隐形冠军”企业培育计划和高新技术企业“倍增”计划。鼓励发展研究型创新型企业和“独角兽”企业，建设跨境创新加速器，力争引进具备重大产业化潜力的国际优质创新企业20家以上。充分发挥人才引领作用。推进人才生态最优城市建设，深入落实“国千”“万人计划”“省千”和市“521”“高层次人才特殊支持”等引才育才计划，实施“名校名院名所”建设工程，加快西湖大学、浙江工程师学院、北京大学信息技术研究院、北京航空航天大学杭州创新研究院和研究生院、奥克兰大学中国创新研究院等重点载体建设，加强与中国科学院大学等国内外一流大学的合作，大力支持之江实验室、浙江大学超重力离心模拟与实验装置、阿里达摩院建设，推进中国美院良渚校区、浙江理工大学余杭国际校区建设，深化国际人才创业创新园建设，吸引更多海内外各类人才及团队来杭创新创业。弘扬杭州工匠精神，建设高技能人才队伍。充分发挥平台保障作用。推进国家双创示范基地建设，提升特色小镇、众创空间、孵化器等创新载体建设水平，新增省级高新特色小镇2家、市级以上众创空间20家，建设省级产业创新服务综合体2家，高水平建设杭州硅谷协同创新中心。加快城西科创大走廊、城东智造大走廊、杭州临空经济示范区、之江文化产业带等建设，更好发挥大江东产业集聚区、杭州高新区（滨江）、杭州经济开发区等重大平台在推动创新发展、做强实体经济和制造业方面的重要作用。

深化供给侧结构性改革，以“破、立、降”为抓手，加快动能转换和结构调整。大力破除无效供给、坚决淘汰落后产能。严格执行质量、环保、能耗、安全等法规标准，加大违法违规行为查处力度，完成30家“僵尸企业”处置，淘汰100家企业落后产能，大力整治脏乱差小作坊和无证照、无安全保障、无合法场所、无环保措施“四无”企业。大力培育新动能、加快发展新产业。进一步发展大数据云计算、数字安防、信息软件、电子商务、智能制造等优势产业，推进5G、IPv6设施应用，巩固扩大数字经济在全国的领跑优势。发展壮大生物医药、集成电路、物联网、新能源汽车、高端装备制造等战略性新兴产业，加快培育人工智能、虚拟现实、区块链、量子技术、商用航空航天等未来产业。做大做强数字内容、动漫游戏、影视创作、演艺等文创特色产业。积极推进军民融合发展，建设军民融合创新研发转化平台，培育军地科技协同创新孵化基地。大力推进传统产业改造升级。突出纺织、服装、化工等12个重点行业，聚焦化纤、造纸两个省级改造试点，大力推进数字驱动、两化融合、智能制造。支持丝绸、中医药、茶叶、食品饮料等传统优势产业和历史经典产业发展。推广应用工业互联网，实施企业上云4万家、机器换人450项、工厂物联网100项。加强质量建设，新增“浙江制造”标准50项、品牌10个。继续降低实体经济成本。严格执行减负降本政策，进一步削减涉企办事费用，全面推进企业分类综合评价，逐步实施资源要素差别化政策，降低鼓励扶持类产业用水、用电、用气、物流、融资成本，为实体经济发展创造良好环境。

坚持以有效投资提高供给质量、以消费升级带动产业升级、以服务业发展更好满足多元需求。发挥投资对优化供给结构的关键性作用。确保交通投资、生态环境和公共设施投资、高新技术产业投资、民间投资均增长10%以上。把产业投资作为重中之重任务来抓，加大重大产业项目招引力度，围绕主导产业发展，强化“一把手”招商，实施市长和区县（市）长项目工程制度，每个区县（市）至少招引一个投资20亿元以上的产业大项目。增强消费和现代服务业的拉动作用。完善促进消费的体制机制，营造放心舒心消费环境，积极培育服务消费、信息消费、绿色消费、时尚消费、品质消费、农村消费等新兴消费。推动商贸业转型发展，打造购物新天堂。大力发展工业设计、现代物流、科技研发、会展服务等生产性服务业，积极发展体育健身、健康养老等特色服务业，提升服务业集聚区发展水平。

（三）聚焦实施拥江发展战略，着力提升区域统筹协调发展水平。“拥江发展”是杭州落实党的十九大关于实施区域协调发展、可持续发展战略和省委关于大湾区大花园大通道大都市区建设的主要载体，是我市优化市域空间布局、推进市域协调发展的重大举措。今年是拥江发展实质性推进第一年，要按照《关于实施“拥江发展”战略的意见》，积极实施“四年行动计划”，扎实打造自然生态带、魅力文化带、公共景观带、综合交通带、现代产业带和宜居城市带，推动形成以城带乡、以东带西市域协调发展新格局。

大力推进规划统筹引领。坚持多规融合、两规合一，高质量推进新一轮城市总体规划和土地利用总体规划编制工作，以全球视野对标先进、读懂杭州，做到对历史、文化、底蕴、特色“守得住”，对生态保护红线、永久基本农田、城镇开发边界“控得牢”，对杭州未来发展“望得远”。强化规划的市级统筹管理，优化提升“多中心、网络化、组团式、生态型”市域空间格局。围绕钱塘江综合保护与利用，制定实施《杭州市钱塘江两岸综合保护与利用导则》等规范性文件，加强钱塘江岸线管控，加快生态修复。制定实施杭州市拥江发展战略规划，加强重点地区和重要节点城市设计，高水平规划建设钱江新城二期、钱江世纪城、奥体博览城、望江金融科技城、之江新城、大江东核心区、下沙副城、富阳江南-东洲新城等区块，协调推进桐庐、建德、淳安等县（市）的新城建设。

大力推进市域一体发展。以“拥江发展”理念下好“全市一盘棋”。西湖是杭州的“根”与“魂”，要持续提升保护水平，整治景中村等问题，加强景区管理，放大溢出效应。以建设城西科创大走廊为重要载体，强化未来科技城、青山湖科技城、紫金港科技城等建设，提高城西地区发展水平。以建设大运河文化带（杭州段）为牵引，深化实施运河综合保护工程，全面推进大城北地区改造，加快武林新城、运河新城、杭钢新城、良渚新城建设，加强路网等基础设施建设的统筹协调，使大城北成为我市发展的重要增长极。持续推进中心城区非核心功能疏散，加快副城组团基础设施和公共服务设施建设，推进萧山余杭富阳临安4区与杭州主城区深度融合发展；深化区县（市）协作、联乡结村工作，更好发挥杭州市区对桐庐淳安建德3县（市）的辐射带动作用，在更高层次上提升城乡融合发展水平。

要以2022年亚运会召开为重要节点，大力推进交通、环境、亚运基础设施“三大建设行动”和城中村改造攻坚、小城镇环境综合整治“两大整治行动”，一年接着一年干，一以贯之，久久为功。实施交通基础设施建设行动。加快萧山国际机场扩容项目建设，拓展加密国际航线。实施《杭州铁路枢纽规划(2016-2030年)》，开通杭黄铁路，开工建设铁路杭州西站、商合杭铁路湖州至杭州段等铁路项目，推进沪乍杭及连接线等铁路前期工作，谋划建设杭州至武汉高铁。加快建设地铁二期和杭富、杭临等轨道交通项目，全面开工地铁三期项目，积极推进铁路杭州西站至萧山机场轨道快线项目前期，完成地铁建设投资330亿元。建成文一路地下通道、望秋立交、东湖快速路北延、秋石快速路北延、之浦路三期，新开工文一路西延、艮山快速路等项目。推进杭绍甬高速杭州至绍兴段等项目前期工作，提升改造沪杭甬高速部分路段，续建绕城高速西复线、千黄高速，完成杭金衢高速杭州段拓宽工程。加快运河二通道建设。新建停车泊位45000个。江东大桥实现免费通行。推进地下综合管廊建设，开发利用地下空间500万平方米。持续推进交通治堵工作，深化公交都市创建，实施“城市数据大脑2.0版”，提升智慧交通管理水平。实施环境基础设施建设行动。抓好中央环保督察整改落实工作，实施全市固废处置能力建设行动计划，加快天子岭餐厨与厨余二期等项目建设，推进循环产业园区、分类减量综合体、第三固废处置中心以及飞灰协同处置等项目建设。新建及改造污水管网110公里，完成七格污水处理厂四期土建工程，建成投用临平净水厂等2个项目，续建之江净水厂、临江污水处理厂二期等3个项目，开工建设余杭污水处理厂四期等3个项目。实施亚运基础设施建设行动。高标准推进亚运会场馆设施建设，全面开工亚运村和5个新建场馆，竣工奥体中心主体育场、网球中心、杭师大仓前校区体育馆等场馆，基本完成改造场馆项目前期，研究确定并尽快组织实施新增项目比赛场馆及训练、备用场馆。实施城中村改造攻坚行动。继续推进以主城区为重点的城中村改造，实施改造整治、安置房建设、回迁安置、公共配套设施建设四个行动计划，完成56个村整村拆迁。深入推进城镇危房治理改造。实施小城镇环境综合整治行动。深入实施规划设计引领等专项行动，整治项目总体形象进度达到80%以上，打造一批有特色有亮点的示范小城镇，小城镇环境综合整治完成的乡镇累计达到105个。深入推进“三改一拆”，完成“三改”1950万平方米，拆除违法建筑1400万平方米。

（四）聚焦实施乡村振兴战略，着力提升农业农村现代化水平。实施乡村振兴战略，是新时代“三农”工作的总抓手，也是我市打造美丽乡村升级版、加快农业农村现代化的重要举措。认真贯彻落实《中共中央国务院关于实施乡村振兴战略的意见》，按照产业兴旺、生态宜居、乡风文明、治理有效、生活富裕的总要求，制定加快农业农村现代化建设行动方案，加快美丽田园、美丽庭院、美丽村庄建设，推动形成城乡融合发展新格局，争创乡村振兴示范区。

深化农村改革增活力。统筹推进农村土地制度和集体产权制度改革，做好承包地、宅基地、集体建设用地“三块地”文章，推动资源要素更多流向农村，全面激活农村发展。积极探索承包地“三权”分置有效方式，加快农村土地承包经营权确权登记颁证工作进度，扩大土地流转规模，发展适度规模经营。按照讲求质量、边界可控、主体清晰、农民得益原则，因地制宜，分批次推进农村全域土地综合整治，并与农业发展、村庄整治、集体经济发展、历史村落保护等有机结合起来，实现耕地集中连片保护，建设用地集约高效，生态环境美丽宜居。深化宅基地管理改革，完善乡村规划建设机制，以“带方案审批”为抓手加强农房规划建设管理，提升村庄建设管理水平。深化耕地占补平衡管理改革，建立补充耕地指标有偿调剂机制，激活农村资产资源，撬动农村集体经济发展。

加快农村产业发展强动力。深化农业供给侧结构性改革，坚持质量兴农、绿色兴农、效益优先，推进现代农业园区建设，积极培育农业龙头企业、农民专业合作社、家庭农场、种养大户等新型经营主体和社会化服务主体，加强农业科技推广和应用，大力发展生态品质农业、智慧设施农业、现代都市农业。确保粮食生产、供应和储备能力，保障粮食安全，加强菜篮子工程建设。推进“互联网+现代农业”，推进田园综合体建设，大力发展农村电商，培育壮大民宿经济、乡村旅游、养生养老、运动健康等新产业新业态，促进农村一二三产业融合发展。实施下姜村及周边地区振兴发展等规划。完善农田水利基础设施，新增高效节水灌溉面积2.78万亩，改善灌溉面积4万亩；整治高坝、屋顶山塘80座，除险加固小型水库15座。

强化农村环境整治添魅力。加强农村突出环境问题综合治理，持续推进拆违治危，深化农户庭院、农村各类杆线等整治，建设“四好农村路”，广泛开展“厕所革命”“垃圾革命”，启动打造精品村70个以上，继续推进精品线路、风情小镇和历史文化村落建设。开展农村移风易俗活动，塑造淳朴文明的良好乡风。强化农村基层基础工作，加强农村基层干部队伍培养、配备、管理和使用，健全自治、法治、德治相结合的乡村治理体系，走乡村善治之路。

（五）聚焦打好三大攻坚战，着力跨越发展稳定重要关口。坚决打好防范化解重大风险、精准帮扶、污染防治的攻坚战，加大难点问题和薄弱环节破解力度，强基固本，努力保障高水平全面小康目标的实现。

坚决防范化解重大风险。有效防控金融风险，加强重点行业、地区和企业“两链”风险管控，保持不良贷款和关注类贷款“双降”。严厉打击违法违规金融活动，加快建设金融风险“天罗地网”监测防控系统，着力完善安全防线和风险监督预警机制。把握政府性投资项目的建设时序和规模，优化投融资结构，加强统筹安排。实施政府债务计划管理，控增量、减存量，有效防范政府债务和财政风险。加强自然灾害防范应对。推进地质灾害隐患点除险安居工作。加快西险大塘提标加固、城西南排通道等工程前期工作，推进杭嘉湖南排八堡排水、五堡排涝泵站、萧山区蜀山片外排大治河泵站、大江东片外排东湖调蓄湖等工程建设，提升城乡防洪标准。加强城市安全管理，争创国家安全发展示范城市。严格落实安全生产责任制，强化企业主体责任，加大对重点地区和危化品、矿山、建筑施工、道路交通等高风险领域风险排查整治力度，推广居住出租房屋“旅馆式”管理，严格落实出租房及高层建筑、人员密集场所消防安全管理，坚决防范事故发生。全面推广品质食品示范超市，深化放心餐饮店创建，争创国家食品安全示范城市。全面实施居住证积分管理政策。深化基层治理体系“四个平台”建设，完善在线矛盾纠纷多元化解平台和社会心理服务体系，提高社会治理的社会化法治化智能化专业化水平。加强社会治安综合治理，织密社会治安立体防控网，推进“雪亮工程”，扎实开展扫黑除恶专项行动，深化平安创建，努力打造全国最安全城市。

深入推进精准帮扶。实施消除集体经济相对薄弱村三年行动计划，盘活集体资产，增强集体经济造血功能，年经营性收入达到市定目标的村占总村数的比例达到80%。深入实施低收入农户全面小康行动计划，低收入农户人均收入增长10%以上；以就业困难人员、城镇零就业家庭、持证残疾人等为重点，深化就业援助和救助行动，强化农村留守儿童关爱保护。深入实施山海协作工程，继续做好贵州黔东南州、湖北恩施州、新疆阿克苏、西藏那曲等扶贫协作和对口支援工作。

持续推进污染防治。打好蓝天保卫战，推动PM2.5浓度持续下降、空气质量优良天数持续上升。全面推进全市域“清洁排放区”建设，以调整优化产业结构为重点，淘汰关停一批化工、印染、造纸等高污染、高耗能企业，大幅度削减主要污染物排放量；制订实施挥发性有机物、锅炉大气污染物等地方排放标准；推进国Ⅲ柴油货运车、混凝土车辆、渣土运输车、环卫车淘汰和清洁化更新；加强建设工地扬尘标准化管理；实施预拌混凝土企业绿色转型升级三年行动计划。深化“五水共治”，打造全市域污水“零直排区”，开展“美丽河湖”创建工作，推进农村污水处理设施改造提标工程，巩固提升“清三河”和剿灭劣V类水成果。深化生活垃圾“三化四分”，着力推进智慧垃圾分类，累计创建省级垃圾分类示范小区60个、市级示范小区200个。实施土壤污染防治行动计划，强化土壤污染场地监管和修复。

加大生态系统保护力度，完善生态补偿机制，编制市级自然资源资产负债表。优化能源结构、转换用能方式，推广合同能源管理、电力需求侧管理等节能新机制。发展装配式建筑，推广住宅全装修，开展各类绿色创建行动。

（六）聚焦优化公共服务供给，着力提升民生保障质量。以满足人民群众的美好生活需要为导向，坚持保基本、兜底线、促均衡、优品质，进一步完善公共服务体系，切实保障和改善民生，增强人民群众获得感、幸福感和安全感。

统筹推进社会事业发展，加强优质公共服务均衡供给。坚持教育优先发展。美好生活从美好教育抓起，制定实施“美好教育三年行动计划”，全面构建高水平教育服务体系，促进各级各类教育协调发展。进一步推进义务教育高质量均衡发展，加大教育发展的财政投入，完善中小学和幼儿园布局，加快市域范围内名校集团化建设，加强教育管理，努力解决中小学生课外负担重等突出问题。积极发展学前教育。有计划高质量地设立外籍人员子女学校，推进教育国际化。积极引进国内外一流大学来杭合作办学，支持市属高校提升发展，提高产教融合、校企合作水平。提高就业质量和社会保障水平。深入实施就业优先战略行动、创业带动就业行动、职业技能提升计划，进一步完善职业培训体系。健全劳动报酬正常增长机制，落实体现知识技能价值的收入分配政策。启动实施临安社保一体化工作，进一步完善社会保障体系。加快健康杭州建设。全面落实《“健康杭州2030”规划纲要》，深化医药卫生体制改革，推进高水平医联体建设。健全分级诊疗体系，提高家庭医生签约服务质量，打造智慧医疗服务升级版。加强院前医疗急救体系建设，推进红十字关爱生命工作。做好迎接国家卫生城市复评工作。支持发展托儿机构。巩固养老服务业综合改革试点成果，进一步提升养老服务质量，积极构建养老孝老敬老政策体系和社会环境。大力发展文化体育事业。继续做好良渚古城遗址申遗工作。加强南宋皇城遗址综合保护。推进市群众文化中心、市非遗保护中心、市城市档案中心、县级博物馆建设，启动杭州艺术学校改扩建工程。深化实施百家社区文化行、群文配送、农村电影放映和城市电影惠民等工程，丰富群众文化生活。推进市全民健身中心建设和市体育馆改造提升，新建20处全民健身中心（公园、广场），推进公共体育场馆设施免费或低收费向社会开放。做好2018年第14届国际泳联世界游泳锦标赛（25米）的服务保障工作。加强精神文明建设。深入践行社会主义核心价值观，大力弘扬城市人文精神，持续开展“争做最美杭州人、创建最美文明城”活动。唱响主旋律，做好纪念改革开放40周年等重大主题宣传。深化全民国防教育，增强全民国防意识。完善公共法律服务体系，加大普法力度，加强志愿服务，发展公益事业，进一步提高市民法治素养、道德素质和社会文明程度，让城市更有“温度”。

住房保障、安置房及配套设施建设、区域供水体系建设是现阶段事关全市人民的重大民生工程，必须着力抓紧抓好。要深化住房保障制度改革。按照中央“建立多主体供应、多渠道保障、租购并举的住房制度”改革要求，深入推进国家住房租赁试点工作，健全市场租赁房、人才租赁房、公共租赁住房等多元化保障体系，大力发展住房租赁市场特别是长期租赁市场，确保新增租赁住房占新增商品住房总量30%以上。积极推进蓝领公寓建设，着力解决外来务工人员住房需求。要切实落实安置房及配套设施建设任务。开工建设城中村改造和拆迁安置房1500万平方米，回迁安置1万户；按照打造一流公共服务、公共设施和公共空间的标准，编制实施《杭州市城中村改造配套设施项目建设计划（2018-2020年）》，开工建设教育、卫生、道路、河道等一批配套设施项目。要加快千岛湖配供水工程建设。实现113公里主线隧洞贯通，开工江南线配套工程、推进城北线配套工程建设、全面竣工九溪线配套工程，为2019年形成千岛湖和钱塘江“双水源”供水格局奠定基础。

我们既要抓重大民生工程建设，也要办好群众最关注、最直接、最现实的“关键小事”。根据省市委关于政府民生实事项目人大代表票决制决定，前期市政府从市民群众、“两代表一委员”、市级部门等层面广泛征集，遴选形成12个候选项目。经本次人代会票决，以下10个项目确定为市政府2018年度民生实事项目：

1.按照“四好农村路”要求，提升改造农村公路380公里、实施精品示范公路145公里，完成60条城市道路增亮。

2.完成农村住房改造1.2万户、农村困难家庭危房改造1150户、农村C级危房治理3000户。

3.对全市签约居民中的16种慢性病患者，一次处方医保用药量从4周延长至最多12周；新增公共场所母婴设施120处。

4.在杭州主城区151所小学开展学后托管服务，建成100个市级名师乡村工作室、培训1000名乡村教师。

5.升级完善68个重点社区微型消防站，设置电动自行车集中充放点1000处、电动自行车智能充电口1万个，在全市出租房及工棚等场所安装独立式感烟探测器3万个。

6.积极推进市区既有住宅电梯加装，对符合加装条件的做到“愿改尽改”；电梯安装智慧监管装置1万台以上，实现10万台以上电梯维保透明全覆盖。

7.建设150个农村文化礼堂，建设615个村（社区）公共文化场地WIFI设施、实现全市村（社区）全覆盖。

8.推进“厕所革命”，新建改造提升750座公共厕所（其中：城区公厕100座、涉旅游厕所150座、农村公厕500座）。

9.全市新增城镇就业21万人，帮助失业人员再就业10万人，接收高校应届毕业生7万人。

10.全市持证残疾人免费乘坐市内地铁、公共汽电车和水上公交巴士。

对上述民生实事项目，市政府将加强组织领导、严格责任考核、强化统筹协同、狠抓工作落实，并接受市人大代表和社会各界监督检查，确保实事办好、好事办实。

各位代表，迈入新时代、肩负新使命，要有新气象,更要有新作为。市政府将始终坚持党的领导，按照信念过硬、政治过硬、责任过硬、能力过硬、作风过硬的要求，不断加强自身建设，努力推动各项工作再创新业绩、杭州发展再上新台阶。要做到坚定忠诚。牢固树立“四个意识”，严守政治纪律和政治规矩，坚决维护以习近平同志为核心的党中央权威和集中统一领导，忠诚核心、维护核心、看齐核心。认真领会贯彻上级党委政府和市委的各项决策部署，狠抓各项工作执行落实。要严格依法行政。切实将政府活动全面纳入法治轨道，严格规范公正文明执法，深化行政复议体制改革，以政府依法施政带动全社会尊法守法。认真执行市人大及其常委会决议决定，坚持重大事项向市人大及其常委会报告、向市政协通报制度，自觉接受人大的法律监督、工作监督和政协的民主监督。要切实增强本领。用习近平新时代中国特色社会主义思想持续武装头脑，不断增强学习、政治领导、改革创新、科学发展、依法行政、群众工作、狠抓落实、驾驭风险等八个本领，努力建设高素质专业化干部队伍，加快提升政府治理体系和治理能力现代化水平。要不断改进作风。认真开展“不忘初心、牢记使命”主题教育，忠诚敬业、勤勉为政、担当尽责。大兴调查研究之风，坚持群众路线，锲而不舍解决实际问题。自觉扛起全面从严治党主体责任，坚决同各种不正之风和腐败现象作斗争。严格贯彻中央八项规定及实施细则精神，驰而不息纠正“四风”，自觉接受监察监督，强化审计监督，推动政务公开，严格财经纪律，倡导勤俭办一切事情，建设对党忠诚、人民满意的政府。

各位代表，让我们更加紧密团结在以习近平同志为核心的党中央周围，在习近平新时代中国特色社会主义思想指引下，在省委省政府和市委领导下，大力弘扬“红船精神”，坚定信念、脚踏实地，尽心竭力、勤勉实干，为加快建设独特韵味别样精彩世界名城、不断满足人民群众日益增长的美好生活需要而努力奋斗！
